# Plasma CC16 levels are associated with development of ALI/ARDS in patients with ventilator-associated pneumonia: a retrospective observational study

**DOI:** 10.1186/1471-2466-9-49

**Published:** 2009-12-03

**Authors:** Rogier M Determann, Julian L Millo, Sam Waddy, Rene Lutter, Chris S Garrard, Marcus J Schultz

**Affiliations:** 1 Department of Intensive Care Medicine, Academic Medical Center, Amsterdam, The Netherlands; 2 Laboratory of Experimental Intensive Care and Anesthesiology (L.E.I.C.A), Academic Medical Center, Amsterdam, The Netherlands; 3 Department of Intensive Care Medicine, John Radcliffe Hospital, Oxford, UK; 4 Department of Experimental Immunology, Academic Medical Center, Amsterdam, The Netherlands; 5 Department of Pulmonology, Academic Medical Center, Amsterdam, The Netherlands; 6 HERMES Critical Care Group, Amsterdam, The Netherlands

## Abstract

**Background:**

Despite consensus criteria, diagnosing acute lung injury, or its more severe form acute respiratory distress syndrome (ALI/ARDS) remains challenging. Adding objective measures, such as plasma levels of biological markers could facilitate recognition of ALI/ARDS. This study was designed to assess and compare the diagnostic accuracy of biological markers for ALI/ARDS with ventilator-associated pneumonia (VAP).

**Methods:**

We performed serial measurements of Clara cell protein (CC16), soluble receptor for advanced glycation end products (sRAGE), surfactant protein D (SP-D) and Krebs von den Lungen (KL-6) in plasma of patients with VAP and mechanically ventilated control patients without VAP. ALI/ARDS was diagnosed using the criteria of the North-American European consensus conference.

**Results:**

Thirty-seven patients were enrolled - 22 patients with VAP and 15 control patients. Ten patients with pneumonia met the ALI/ARDS consensus criteria. Control patients never met these criteria. Plasma CC16 had a good diagnostic capacity for ALI/ARDS as shown by the receiver operating characteristic curve with an area under the curve of 0.91 (95% confidence interval (CI) 0.79 - 1.00; *p *< 0.001). Identification of ALI/ARDS patients by sudden increases in plasma CC16 of 30% or more yielded a sensitivity of 90% and a specificity of 92%. Of note, levels of CC16 increased 2 days before ALI/ARDS diagnosis. A cut-off level of 50 ng/ml SP-D yielded a specificity of 100% while the sensitivity was 70%. The area under the curve for SP-D was 0.80 (95% CI 0.58 - 1.00; *p *= 0.02). The diagnostic accuracies of KL-6 and sRAGE were low.

**Conclusion:**

Plasma CC16 seems a potential biological marker for ALI/ARDS in patients with VAP. Plasma levels of sRAGE, SP-D and KL-6 have limited discriminative power for diagnosing ALI/ARDS in VAP.

## Background

Intubated and mechanically ventilated patients are at risk for ventilator-associated pneumonia (VAP) [1]. Development of acute lung injury (ALI) or its more severe form, acute respiratory distress syndrome (ARDS) decreases the chance of survival [2]. Early and adequate recognition of ALI/ARDS is mandatory for intensive care physicians to take sufficient action at the right time (e.g., the use of so-called lung-protective mechanical ventilation [3], and refinement of fluid management [4]). In today's intensive care practice, ALI/ARDS is diagnosed by means of the North-American European consensus conference (NAECC) criteria [5]. However, diagnosing ALI/ARDS remains challenging, at least in part due to personal interpretation of these criteria [6]. In addition, use of higher levels of positive end-expiratory pressure can improve both the PaO_2_/F_i_O_2_-ratio and abnormalities on chest radiographs to the extent that the patients no longer fulfill the ALI/ARDS criteria (per definition) [7].

Biological markers may facilitate the recognition of ALI/ARDS as they are objectively obtained and not subject to personal interpretation. Many proteins involved in the pathophysiology of ALI/ARDS have been suggested as biological markers, including Clara cell protein (CC16) [8], soluble receptor for advanced glycation end products (sRAGE) [9], surfactant protein D (SP-D) [10] and Krebs von den Lungen (KL)-6) [11]. CC16 is a small 16 kDa protein and is the main secretion product of Clara cells located in the terminal airways [8]. sRAGE is a membrane-bound protein that is strongly expressed by alveolar type I cells [12]. SP-D is produced and secreted by alveolar type II cells as well as Clara cells [13] and KL-6 is expressed by type II cells in the lungs [14]. During pulmonary inflammation, proteins bound to the alveolar epithelial membrane (sRAGE and KL-6) are released in the alveolar space. It is suggested that the membrane-bound proteins are separated from the membrane as a result of cell injury and accumulate in the epithelial lining fluid. In line with this, increased concentrations of KL-6 and sRAGE in epithelial lining fluid of patients with ALI/ARDS have been observed [9,11]. Furthermore, it is postulated that due to increased permeability of the alveolocapillary membrane these proteins and the secretory proteins CC16 and SP-D, leak into the circulation. Indeed, increased plasma levels of CC16, surfactant proteins, sRAGE and KL-6 have been reported in patients with ALI/ARDS [9-11,15,16]. Moreover, plasma CC16 and SP-D levels have prognostic significance in patients with ALI/ARDS [15,16].

While many studies have focused on the prognostic value of these proteins, the diagnostic value for ALI/ARDS has not been investigated in critically ill patients at risk for ALI/ARDS. We measured plasma levels of CC16, sRAGE, SP-D, and KL-6 in intubated and mechanically ventilated patients who were at risk for VAP and determined the diagnostic accuracy of these proteins for the diagnosis of ALI/ARDS in patients with VAP. The present study was primarily designed to study CC16 and SP-D. After recent reports on sRAGE and KL-6 as new potential markers for ALI/ARDS [9,11] we decided to compare these with CC16 and SP-D. Part of the results of this study have been previously reported in the form of an abstract [17].

## Methods

### Study population

A single-center retrospective observational study was conducted between 2001 and 2003 in the intensive care unit of the John Radcliffe Hospital in Oxford, UK. Although the study protocol was performed prospectively, the present study on diagnostic accuracy was designed retrospectively. Patients ≥18 yrs of age who were intubated and mechanically ventilated with an expected duration of mechanical ventilation of ≥72 hrs were included in the study. Immunocompromised patients were excluded. In view of the observational nature of the study, the relatively small volumes of blood sampled (5 ml on alternate days, see further), and the impossibility of obtaining informed consent prospectively, the investigators requested that neither retrospective patient consent nor formal assent from relatives be required. The study protocol allowed for future measurements for retrospective analyses. The complete study was performed in compliance with the Helsinki Declaration. Both the prospective study protocol and the retrospective analyses were approved by the Central Oxford Research Ethics Committee and the need for informed consent was waived for both the prospective collection and the retrospective analyses.

### Diagnostic criteria for VAP

VAP was suspected when a new or progressive infiltrate was present along with at least two of the following signs and symptoms: a) purulent respiratory secretions; b) fever, defined as body temperature ≥38°C, or hypothermia defined as body temperature ≤35°C; and/or c) leukocytosis defined as white blood cell count ≥10,000/mm^3 ^or leucopenia with total white blood cell count < 4500/mm^3 ^or > 15% immature neutrophils (bands) regardless of total peripheral white blood cell count. Diagnosis of VAP had to be substantiated by positive culture results of blindly obtained bronchoalveolar lavage fluid. Non-directed bronchoalveolar lavage (NBL) fluid is routinely obtained in the John Radcliffe hospital as part of microbiological surveillance [18]. If VAP was suspected antimicrobial treatment was started if warranted. However, definite diagnosis of VAP was made retrospectively if the clinical, radiological and microbiological data were deemed consistent with a diagnosis of VAP by two independent senior clinicians as described previously [19]. Patients without infectious lung pathology by clinical and radiological criteria who were intubated and mechanically ventilated for at least 5 days served as a control group.

### Diagnostic criteria for ALI/ARDS

ALI/ARDS was diagnosed by the NAECC-criteria [5].

### Data collection

Baseline characteristics (including age, gender, duration of intubation and mechanical ventilation before study enrolment, and prior antimicrobial therapy) and admission diagnosis were recorded for each patient. In addition, respiratory parameters (tidal volume size, level of positive end - expiratory pressure, levels of peak airway pressure) and infectious parameters, as well as the multi-organ dysfunction score (MODS), the clinical pulmonary infection score (CPIS) and lung injury score (LIS) were recorded daily [20-23].

### Sampling and processing

From the day of initiation of mechanical ventilation, blood was collected in tubes coated with ethylenediamine tetraacetic acid each alternate day. The tubes were centrifuged at 1500 × *g *for 10 minutes at 4°C. The supernatant was collected and stored at -80°C.

### Measurements

Levels of CC16 measured with an enzyme-linked immunosorbent assay (ELISA). In short, 96 well plates were coated with 25 ng monoclonal anti-human CC16 antibody AY1E6 (HyCult, Uden, The Netherlands). Calibrator (Biovendor, Heidelberg, Germany) and samples were diluted as appropriate and incubated for one hour. Then the plates were washed and incubated for one hour with 10 ng polyclonal biotinylated anti-human CC16 detection antibody A0257 (Dako, Glostrup, Denmark). After washing, streptavidin labeled with poly-horseradish peroxidase (Sanquin, Amsterdam, The Netherlands) was added and incubated for 30 minutes. Finally, after three washes, 100 μl sodium-acetate buffer (0.11 mol/L, pH 5.5) containing 100 μg/ml tetramethylbenzidine and 0.003% H_2_O_2 _was added and the color reaction was stopped by 2 M H_2_SO_4_. The detection limit of the assay was 10 pg/ml and the recovery of CC16 in plasma was 91-98%. Levels of SP-D and sRAGE were measured as with an ELISA as described previously [24]. Levels of KL-6 were measured with an enzyme immunoassay (Sanko-Junyaku Co., Ltd, Tokyo, Japan) according to the instructions of the manufacturer. All measurements were made in duplicate.

### Statistical analysis

Results are expressed as means ± standard deviation (SD) for normally distributed data or as medians with interquartile ranges (IQR). To compare baseline differences between control patients and VAP patients the Student's *t *test was used or the Mann Whitney U test as appropriate. Proportions were compared using the chi-square test with Yates correction or Fisher's exact test when necessary. Spearman's rho was used to determine the linear relationship between plasma levels of biological markers and LIS. To study changes over time in control patients and patients developing VAP, serial data were analyzed using linear mixed models. To compare differences between control patients and VAP patients, the highest levels observed in control patients were compared with the levels observed on the day of diagnosis of VAP. Differences between VAP patients with or without ALI/ARDS were analyzed by adding presence of ALI/ARDS as a factor into the linear mixed model.

As a next step we investigated the diagnostic accuracy of plasma levels of the pulmonary proteins for the diagnosis of ALI/ARDS in patients with VAP. In order to study whether the protein levels were diagnostic of ALI/ARDS we constructed receiver-operating characteristic (ROC) curves with the values measured on the day of VAP diagnosis. Cut-off values were selected to calculate sensitivities and specificities.

All analyses were performed using SPSS version 14.02 (Chicago, IL, USA). A *p*-value < 0.05 was considered statistically significant.

## Results

### Patients

Thirty-seven patients were enrolled of which 22 patients developed VAP. Fifteen patients did not develop VAP and served as controls. VAP was diagnosed at median 6 [IQR 5-12] days after initiation of mechanical ventilation. Of VAP patients, 10 patients developed ALI/ARDS at the time of VAP diagnosis, or within 2 days. There were no control patients who developed ALI/ARDS. Eight VAP patients and 1 control patient died.

Baseline characteristics and admission diagnosis are presented in table 1. At study entry (i.e., prior to development of VAP), the MODS, LIS and CPIS were higher in patients who developed VAP as compared to control patients. There were no differences between VAP patients who developed ALI/ARDS and VAP patients who did not develop VAP.

**Table 1:** Characteristics of the study population at study enrolment

	Patients with VAP		Patients without VAP (controls)
		
	patients developing ALI/ARDS	patients not developing ALI/ARDS	
	**(n = 10)**	**(n = 12)**	**(n = 15)**

Age (yrs)	67 ± 17	64 ± 13	56 ± 14
Male gender (n, %)	7 (70%)	10 (83%)	8 (67%)
APACHE II-score	22.4 ± 6.7	23.7 ± 4.2	19.7 ± 9.6
MODS	10.8 ± 3.8	10.8 ± 3.0	7.2 ± 3.2*
LIS	1.9 ± 0.8	1.7 ± 1.0	1.1 ± 0.7
CPIS	5.5 ± 1.8	6.7 ± 1.4	4.9 ± 1.4*
Tidal volume (ml)	550 ± 90	585 ± 110	590 ± 130
Tidal volume per kg IBW (ml/kg)	6.6 ± 1.8	7.8 ± 1.6	7.7 ± 1.7
Smoking history	7 (70%)	5 (42%)	7 (47%)
Bronchial asthma	0 (0%)	1 (8%)	0 (0%)
CC16 (ng/ml)	30 [10 - 36]	14 [8.1 - 26]	6.5 [3.6 - 20]
sRAGE (pg/ml)	862 [245 - 1582]	830 [499 - 1527]	560 [350 - 847]
SP-D (ng/ml)	12 [3.7 - 28]	17 [5.1 - 32]	14 [10 - 23]
KL-6 (U/ml)	292 [183 - 495]	279 [161 - 470]	283 [164 - 377]
Admission diagnosis			
Abdominal sepsis	1	2	3
Abdominal aortic aneurysm		1	
Aspiration pneumonia	1	1	
Cardiac arrest	0	2	
Cardiac surgery	3	4	2
Intracranial hemorrhage			2
Meningitis	1	1	
Near drowning	1		
Fasciitis necroticans			3
Pancreatitis	1		2
Trauma	2	1	3

Abbreviations: APACHE, acute physiology and chronic health evaluation; MOD, multiple organ dysfunction; SOFA, sequential organ failure assessment; LIS, lung injury score; CPIS, clinical pulmonary infection score; IBW, ideal body weight; CC16, Clara cell protein; sRAGE, soluble receptor for advanced glycation end products; SP-D, surfactant protein D; *statistical significance (p < 0.05 in post-hoc analysis) of controls vs. VAP patients developing ALI/ARDS and vs. VAP patients not developing ALI/ARDS.

**Figure 1:**
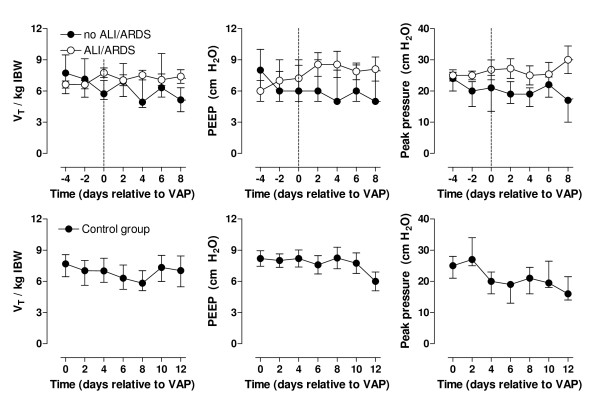
**Respiratory data in VAP patients and control patients**. Tidal volume (V_T_) per kilogram ideal body weight (IBW), positive end-expiratory pressure and peak airway pressure in patients developing ventilator-associated pneumonia (upper graphs) and control patients (lower graphs).

Ventilation data are shown in figure 1. Tidal volumes, levels of PEEP and peak airway pressures at study enrollment were comparable for VAP patients and control patients, and did not change significantly over time. However, levels of PEEP and peak airway pressures increased in patients who developed ALI/ARDS, and were significantly higher after 2 days (p < 0.01 and p < 0.01).

Microbiology data are given in table 2. VAP was caused by Gram-negative pathogens in 13 patients and by Gram-positive pathogens in 9 patients. ALI/ARDS was diagnosed in 7 patients with Gram-negative VAP and in 3 with Gram-positive VAP (p = 0.34).

**Table 2:** Isolated pathogens in patients with ventilator-associated pneumonia

	Gram-positive	Gram-negative
*Escherichia coli*		7
*Pseudomonas aeruginosa*		3
Methicillin-sensitive *Staphylococcus aureus*	2	
Methicillin-resistant *Staphylococcus aureus*	5	
*Streptococcus pneumoniae*	1	
*Enterococcus faecalis*	1	
*Klebsiella pneumoniae*		1
*Morganella morganii*		1
*Haemophilus influenzae*		1

### Plasma levels of pulmonary proteins with VAP

**Figure 2:**
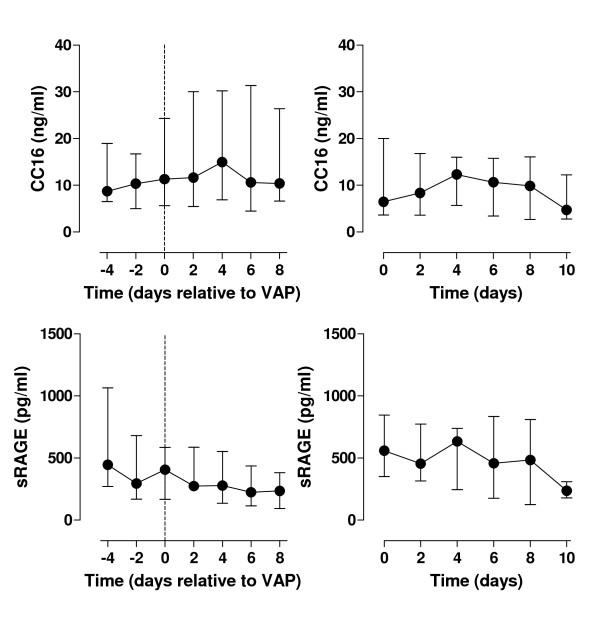
**CC16 and sRAGE levels in VAP patients and control patients**. Plasma levels of Clara cell protein (CC16) and soluble receptor for advanced glycation end products (sRAGE) in patients who developed ventilator-associated pneumonia (left graphs) and mechanically ventilated control patients (right graphs). In the left graphs day 0 represents the day of ventilator-associated pneumonia diagnosis. In the right graphs day 0 represents the day of start of mechanical ventilation.

**Figure 3:**
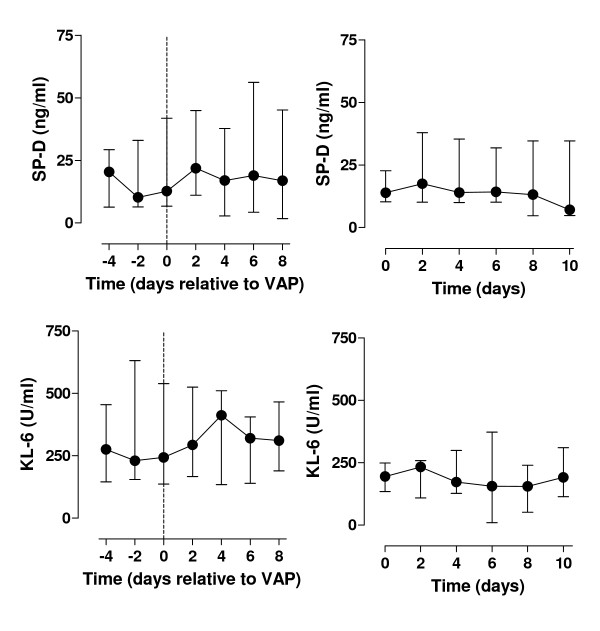
**SP-D and KL-6 levels in VAP patients and control patients**. Plasma levels of surfactant protein D (SP-D) and Krebs von den Lungen (KL-6) in patients who developed ventilator-associated pneumonia (left graphs) and mechanically ventilated control patients (right graphs).

At study entry, plasma levels of all pulmonary proteins were comparable between VAP patients and controls (table 1), although there was a trend for a higher CC16 level in patients who developed VAP. Serial data are shown in figure 2 and 3. Figure 2 shows plasma levels of the smaller proteins CC16 and sRAGE, figure 3 shows plasma levels of the larger proteins SP-D and KL-6. Overall, plasma levels of pulmonary proteins did not change over time, neither in VAP patients nor in control patients.

### Plasma levels of pulmonary proteins with ALI/ARDS

**Figure 4:**
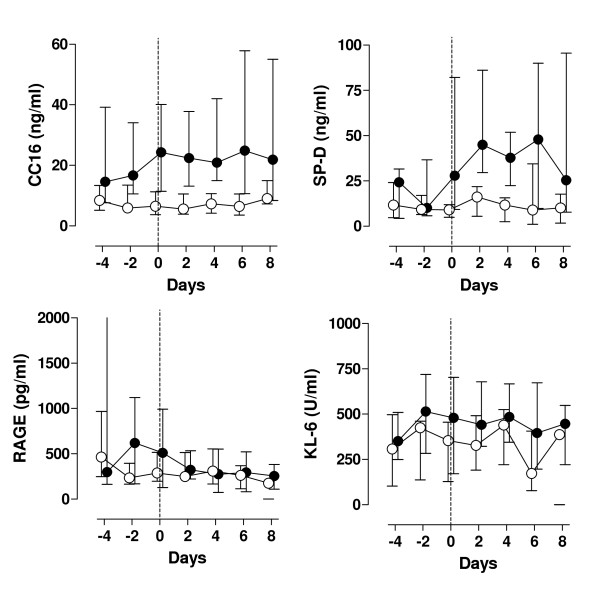
**Biomarker levels in VAP patients with and without ALI/ARDS**. Plasma levels of CC16, sRAGE, SP-D and KL-6 in patients developing ventilator-associated pneumonia. Open circles: patients without ALI/ARDS after onset of ventilator-associated pneumonia, closed circles: patients who progressed to ALI/ARDS at or after onset of ventilator-associated pneumonia.

While plasma levels of CC16 and SP-D remained constant in VAP patients who did not develop ALI/ARDS, plasma levels of these pulmonary proteins increased in patients who eventually developed ALI/ARDS. Patients with ALI/ARDS had significantly higher plasma levels of CC16 and SP-D (linear mixed models, P = 0.003 and p = 0.01 respectively, figure 4). Plasma CC16 levels became significantly different between the ALI/ARDS and the non-ALI/ARDS group 2 days before diagnosis of VAP. No differences were found for plasma sRAGE and KL-6 levels, although there was a trend for a difference in plasma KL-6 levels (p = 0.07).

Plasma CC16 levels correlated with the LIS (Spearman's rho 0.32, p < 0.001). Plasma SP-D, KL-6 and sRAGE levels showed no correlation with the LIS.

### Receiver operating characteristic (ROC) curves

**Figure 5:**
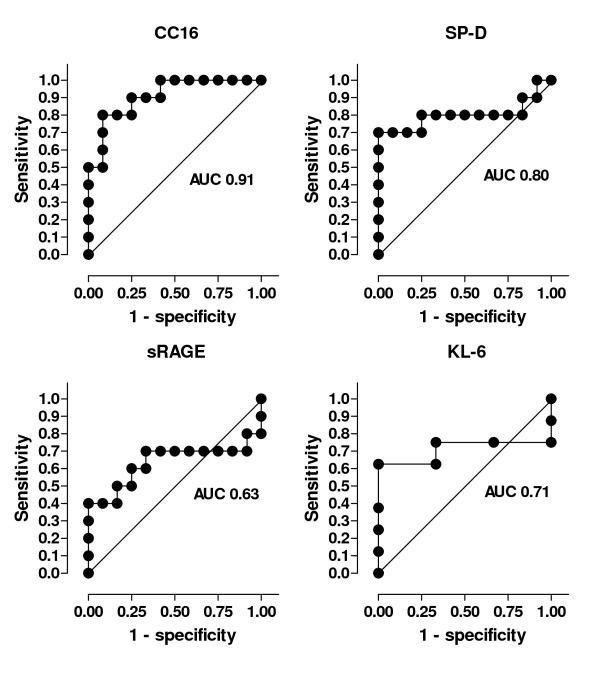
**ROC curves**. Receiver operating characteristic curves of CC16, sRAGE, SP-D and KL-6 for the diagnosis of ALI/ARDS on the day patients developed ventilator-associated pneumonia. AUC area under the curve.

To determine the diagnostic accuracy of plasma levels of these proteins for the diagnosis of ALI/ARDS we constructed ROC curves (figure 5). For this analysis, plasma CC16 levels on the day of ALI/ARDS diagnosis were used and compared to the highest levels observed in VAP patients without ALI/ARDS. CC16 had excellent diagnostic capacity as shown by an area under the curve (AUC) of 0.91 (95% confidence interval (CI) 0.79 - 1.00; p = 0.001; Fig. 5). A level of 18 ng/ml or higher was diagnostic of ALI/ARDS with a sensitivity of 80% and a specificity of 92%. This cut-off level yielded a sensitivity of 80% as one ALI/ARDS patient had a plasma level of 9 ng/ml and one ALI/ARDS patient had a plasma level of 14 ng/ml on the day of diagnosis of ALI/ARDS.

As the number of Clara cells in the bronchioli and plasma CC16 levels are chronically lowered in smokers and in asthma patients [25,26], we analyzed whether ALI/ARDS patients would be better identified by acute increases in CC16 levels and not by absolute values. For this analysis we considered an increase of 30% or more on the day of ALI/ARDS diagnosis as positive. With this approach, only one patient was falsely missed (sensitivity 90%) while the specificity remained 92%. In our cohort, the positive predictive value was 90% and the negative predictive value 92%.

The AUC for SP-D was 0.80 (95% CI 0.58 - 1.00; p = 0.02). None of the VAP patients without ALI/ARDS had a plasma SP-D level of 50 ng/ml or higher (specificity 100%). However, with a cut-off level of 50 ng/ml only 7 patients with ALI/ARDS were identified (sensitivity 70%). An approach identifying ALI/ARDS patients by relative increases did not yield better results (data not shown). The diagnostic accuracy of KL-6 and sRAGE were low as shown by an AUC of 0.71 (95% CI 0.40 - 1.00; p = 0.31) and 0.63 (95% CI 0.37 - 0.90; p = 0.29).

## Discussion

In current clinical practice, diagnosing ALI/ARDS remains a major challenge. Recognizing patients with ALI/ARDS is important so that management strategies such as lung-protective mechanical ventilation using lower tidal volumes and restrictive fluid strategies can be applied [3,27]. Moreover, while clinical studies on new therapies are performed, specific therapies for ALI/ARDS may become available in the near future [28]. In the present study we found plasma CC16 levels to have a good diagnostic accuracy for the diagnosis of ALI/ARDS in patients with VAP.

Two clinical definitions are currently employed to diagnose ALI/ARDS in mechanically ventilated patients: the NAECC definition [5] and the LIS as described by Murray *et al*. [23]. A third definition, as defined by the Delphi criteria has been developed recently [29]. One problem with all three definitions is that they are subject to personal interpretation [6]. Adding objective components to scoring systems might increase their reliability. Many proteins have been proposed as biological markers for ALI/ARDS. A practical problem is, however, that many of these markers have to be measured in epithelial lining fluid or bronchoalveolar lavage fluid which is difficult and laborious to obtain. Blood samples can however be easily drawn from indwelling catheters. Pulmonary proteins that leak to the circulation in a pathophysiological way related to (development of) ALI/ARDS, may serve as biological markers. Previous experimental studies showed plasma CC16 levels to rise in close relation to pulmonary injury [30-32]. To our best knowledge, no reports exist on the diagnostic accuracy of these pulmonary proteins for the diagnosis ALI/ARDS. The present study shows that plasma CC16 levels have excellent diagnostic accuracy and is superior to plasma levels of SP-D, sRAGE and KL-6.

One problem with studying new biological markers is that the measurement techniques used by different study groups are not standardized. In most studies levels of CC16 have been measured by a latex immunoassay while for measuring levels of SP-D many different techniques are employed. Prior to the study, we compared results obtained by our ELISA with results from measurements with the latex immunoassay described by Bernard *et al. *[33]. On 80 samples, the correlation between both measurement techniques was 0.70 and the 95% limits of agreement were 1.6 ± 9.0 ng/ml (data not shown). Therefore, our results can be compared with data from studies that used the latex immunoassay. Due to a different ELISA technique, the absolute levels of SP-D from our study cannot be directly compared with levels observed in other studies. However, the recovery of our SP-D assay was 95 to 105%, from which we conclude that we truly measured SP-D.

As the clinical assessment of both ALI/ARDS [6] and VAP [34] are hampered by the lack of a gold standard the sensitivity and specificity of any scoring system is moderate at best. Despite these limitations, we observed a good diagnostic accuracy for plasma CC16 levels for the presence of ALI/ARDS. Furthermore, our results show an increase in plasma CC16 levels even before ALI/ARDS is diagnosed. Therefore, we speculate that increases in plasma CC16 levels may inform physicians on development of ALI/ARDS. Rising plasma CC16 levels may allow for early adjustments of ventilator settings or additional therapies. This, however, needs to be confirmed in prospective studies.

Studies in human volunteers have shown plasma CC16 levels to increase in settings of subtle sub-clinical lung injury [30,32]. This has been ascribed to the fact that CC16 is a small protein that may leak across the alveolocapillary barrier more easily than larger proteins like SP-D and KL-6. As VAP is characterized by intense pulmonary inflammation, we expected the plasma CC16 levels to be lower in ventilated controls as compared to VAP-patients. Our results point out, however, that plasma CC16 levels in VAP-patients are comparable to those in ventilated patients without VAP. However, most of the control patients in our study had one or more risk factors for ALI/ARDS raising the possibility that these patients were similar from a pathophysiological point of view. Indeed, sepsis, trauma, pancreatitis and fasciitis may evoke low grade lung injury which may explain the comparable plasma CC16 levels between patients with VAP and patients without VAP.

Moreover, mechanical ventilation and pulmonary infection may have comparable effects on leakage of CC16. We previously showed plasma CC16 levels to increase with mechanical ventilation [24]. In that study, patients subjected to low tidal volumes and PEEP and patients subjected to high tidal volumes without PEEP had comparable levels of PEEP resulting in equal levels of pulmonary stretch, and therefore maybe comparable CC16 levels. Animal studies have shown CC16 levels to increase when levels of PEEP or tidal volumes are increased [35]. As tidal volumes in the present study were comparable between patients with and without ALI/ARDS. The higher levels of PEEP in the ALI/ARDS group may have contributed to the sustained increase of plasma CC16 levels.

In patients with ALI/ARDS plasma CC16 levels were three times higher as compared to patients without ALI/ARDS. Furthermore, CC16 levels correlated with the LIS. This is in line with the results of others who found the highest CC16 levels in non-survivors of ALI/ARDS [16]. Patients with the highest grade of lung injury may thus have the highest plasma CC16 levels.

We did not find a strong relation between plasma levels of sRAGE and the development of ALI/ARDS. This is in contrast to earlier reports on elevated sRAGE levels in both epithelial lining fluid or bronchoalveolar lavage fluid and plasma of patients with ALI/ARDS [9,36]. Plasma levels of sRAGE may also be influenced by other factors than lung injury as membrane-bound RAGE is also expressed by the endothelium in the circulation [37]. Moreover, different isoforms of sRAGE have been described and different ELISA techniques may detect different isoforms [9]. We used however the same technique as described by investigators who found a non-significant increase in sRAGE in bronchoalveolar lavage fluid of ALI/ARDS patients [36]. In their analysis, however, sRAGE levels from ALI/ARDS patients were compared with healthy controls, and not intubated and mechanically ventilated patients as in our study. This may have accounted for our negative findings.

## Conclusion

Plasma CC16 seems a promising biological marker for ALI/ARDS in patients with VAP. However, before using CC16 as a biological marker for ARDS in VAP, our results need confirmation in larger study populations. Future studies should investigate new scoring systems for ALI/ARDS using biomarkers levels, including levels of CC16. Finally, as the ventilator settings have become more and more important in patient care, studies that investigate the effect of different ventilator strategies on plasma CC16 levels are also warranted.

## List of abbreviations

ALI/ARDS: Acute lung injury/Acute respiratory distress syndrome; NAECC: North-American European consensus conference; CC16: Clara cell protein; SP-D: Surfactant protein D; sRAGE: Soluble receptor for advanced glycation end products; VAP: Ventilator-associated pneumonia; LIS: Lung injury score; MODS: Multi-organ dysfunction score; SOFA: Sepsis-related organ failure assessment; CPIS: Clinical pulmonary infection score; ELISA: enzyme-linked immunosorbent assay; IQR: interquartile range; ROC: Receiver operating characteristic; AUC: Area under the curve.

## Competing interests

The authors declare that they have no competing interests.

## Authors' contributions

Conception and design by RMD and MJS. Acquisition and assembly of data by JLM, SW, RMD and RL. Analysis and interpretation of the data by RMD and MJS. Preparing the first draft of the article by RMD and MJS. Critical revision of the article for important intellectual content by RMD, JLM, RL, SW, CSG, MJS. Final approval of the article by RMD, JLM, RL, SW, CSG, MJS.

## Pre-publication history

The pre-publication history for this paper can be accessed here:

http://www.biomedcentral.com/1471-2466/9/49/prepub
